# Extended transit compartment model to describe tumor delay using Coxian distribution

**DOI:** 10.1038/s41598-022-13836-4

**Published:** 2022-06-16

**Authors:** Jong Hyuk Byun, In-Soo Yoon, Song Yi Lee, Hyun-Jong Cho, Il Hyo Jung

**Affiliations:** 1 Department of Mathematics, College of Natural Sciences, Pusan National University, Busan, 46241 South Korea; 2 Department of Manufacturing Pharmacy, College of Pharmacy and Research Institute of Drug Development, Pusan National University, Busan, 46241 South Korea; 3 Department of Pharmacy, College of Pharmacy, Kangwon National University, Chuncheon, 24341 South Korea

**Keywords:** Cancer, Chemical biology, Computational biology and bioinformatics, Physiology

## Abstract

The measured response of cell population is often delayed relative to drug injection, and individuals in a population have a specific age distribution. Common approaches for describing the delay are to apply transit compartment models (TCMs). This model reflects that all damaged cells caused by drugs suffer transition processes, resulting in death. In this study, we present an extended TCM using Coxian distribution, one of the phase-type distributions. The cell population attacked by a drug is described via age-structured models. The mortality rate of the damaged cells is expressed by a convolution of drug rate and age density. Then applying to Erlang and Coxian distribution, we derive Erlang TCM, representing the existing model, and Coxian TCMs, reflecting sudden death at all ages. From published data of drug and tumor, delays are compared after parameter estimations in both models. We investigate the dynamical changes according to the number of the compartments. Model robustness and equilibrium analysis are also performed for model validation. Coxian TCM is an extended model considering a realistic case and captures more diverse delays.

## Introduction

Transit compartment models (TCMs) of perturbed tumor growth describe the delay process by which tumors are inhibited by drug administration^1–3^. Apoptosis by drugs does not occur immediately but with a delay. The model consists of two parts, representing proliferating cells and damaged cell parts. Some proliferating cells enter transit processes that describe damaged cells’ delay by drug administration. The assumptions in TCMs are as follows. First, the mortality process is weakly considered before treatment. This indicates that proliferating cells are hardly eliminated without drug administration. To illustrate the cell proliferation process, logistic, exponential, and Gompertz models have been commonly used. Simeoni et al. proposed a new growth model that increases exponentially at the beginning and linearly increases after the threshold^2^. The rate at which proliferating cells enter the damaged phases by the drug is formulated by first-order degradation or the method used in the indirect response models^4^.

The other is that the *n*th discrete compartments express the transit processes damaged by drugs. TCMs have well described the delays such a tumor microenvironment (TME)-driven adaptive mechanism^5,6^. Also, TCMs have been successfully applied to pharmacokinetics & pharmacodynamics (PKPD) to explain the change in dynamical behaviors caused by drugs^7–9^. Existing TCMs account for each transit compartment with the same mean residence time (MRT)^10,11^. When the number of phases representing the damaged cells in the TCMs is *n*, the MRT of each phase is considered as $1/{k_1}$, $k_1$ representing MRT in each transit compartment (thus, total MRT ${n}/{k_1}$). This phase is known as following Erlang distribution^12^.

Our considerations are the development of the extended TCMs regarding dynamical flexibility of the delay process at the drug effect phase. For successful modeling, we considered an age-structured model for the proliferating and damaged cells with the ages *a* depending on exposure by the drug. The model had explicit forms in place of age density using the Erlang and Coxian distributions among the phase-type distributions^13–15^. One of these models is the existing model, called Erlang TCMs. The other is the extended model, called Coxian TCM. Erlang TCM describes the transit processes from one another compartment after drug administration. The latter demonstrates the transit processes and additionally involves a death process in each compartment, expressed by $p_i$. Coxian TCM can reflect the realistic situation, such as different death of the cells of the same ages. This approach shows the flexible delays during treatment. Also, we investigate the heterogeneity of *p*, mean of $p_i$’s, to describe delays in Coxian TCM compared to the number of the compartments. We expect Coxian TCM to be applied to various areas such as pharmacology, infectious diseases, and viral and cell dynamics because the delayed effect of tumors can elaborately be reflected by drug administration.

For model comparison, we conduct an experiment study. Lapatinib is an orally bioavailable, small-molecule TKI approved for use in the treatment of breast cancer patients^16^. This blocks the signal transduction of the PI3K and MAPK pathways, resulting in the induction of cell apoptosis and the inhibition of proliferation^17,18^. A mathematical model for the anti-proliferative activity of lapatinib quantitatively analyzed its cell cycle-dependent cytostatic and cytotoxic effects in 2D-cultured HER2-overexpressing MCF10A cells^8^. More recently, a PKPD model incorporating *In* *vitro* cellular growth dynamics in a 2D-cultured HER1-mutant glioblastoma cell line (SF268), pharmacokinetics, and in vivo glioblastoma growth was developed to optimize lapatinib dosing schedules for the treatment of glioblastoma patients^9^. However, there have been no studies on a PKPD model integrating *In* *vitro* cellular growth kinetics/dynamics in 3D-cultured breast cancer cell lines, in vivo systemic and local tissue pharmacokinetics, and in vivo breast cancer growth of lapatinib.

Our study aims to represent a delayed process in which damaged tumor cells are diminished by drugs. This model includes the transit process of cells and additionally considers a process reflecting the sudden death of the cells using Coxian distribution. Compared with the existing TCM model, this model enables the detailed expression of tumor fluctuations.

## Results

### Derivation of the existing TCM: Erlang TCM

The master equation for the perturbed tumor model shown in “Methods” section is as follows:

1 $$\begin{aligned} {\left\{ \begin{array}{ll} \frac{du}{dt}=k_{in}(u,w)-k_{out}(C,u)\\ \frac{dy}{dt}=k_{out}(C,u)-(k_{out}*f)(t), \end{array}\right. } \end{aligned}$$

associated with $u(0)=u_0$, $y(t)=\int _0^\infty \phi (a,t)dt$, and $w(t)=u(t)+y(t)$. Herein, *f* is a density function, and *u* and *y* are proliferating and damaged cells, respectively. $k_{in}$ and $k_{out}$ represent the growth and mortality functions. From Eq. (1), if *f* is from the point distribution (Dirac delta function), that is, $f(a)=\delta (a-T)$, then

$$\frac{dy}{dt}=k_{out}(C(t),u(t))-k_{out}(C(t-T),u(t-T)),$$

which represents a delay differential equation (DDE). This equation represents that all individuals had the same residence time *T*. Next, we derive an explicit system of Ordinary differential equations (ODEs) using Erlang distribution on density function *f*. This resulting system of ODEs is called Erlang TCM that describes the transition process from one compartment to another^1,19^. Systemically, once a drug is administered, some of tumor cells enter the damaged phases. If they cascade a multiple-step process with a chain of compartments, it demonstrates the delays motivated by the pathway of signal transduction^10^. To induce this TCM from Eq. (1), we consider an age density of $f_n$ in place of *f*, and $f_n$ is given by

$$f_n(a) = k_1 \cdot \frac{(k_1 a)^{n-1}}{(n-1)!}e^{-k_1a},$$

which is a density of Erlang distribution. Because $f_n$ is differentiable, we have the following relations:

$$\begin{aligned} {\left\{ \begin{array}{ll} \frac{df_1(t)}{dt}=-k_1f_1(t)\\ \frac{df_n(t)}{dt}=k_1 (f_{n-1}-f_n(t)),~n\ge 2. \end{array}\right. } \end{aligned}$$

**Figure 1:**
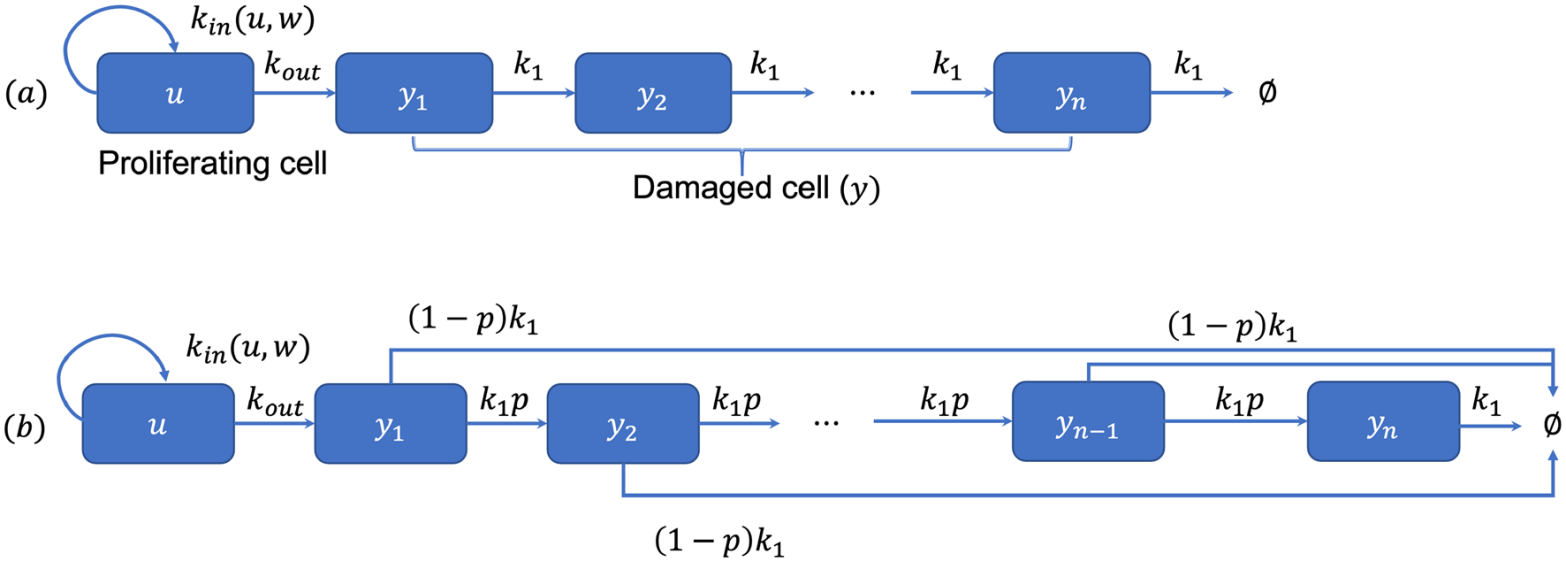
Schematic diagram of TCMs modeled by distributions. *u* and $y_{i}$’s represent the proliferating cell and damaged cell by drug administration. $k_{in}$ and $k_{out}$ represent the growth and mortality. (**a**) Erlang distribution. The transit rate is given by ${k_1}$. (**b**) Coxian distribution. The transit rate is given by ${k_1\cdot p}$. Additional elimination rate $(1-p)k_1$ is derived by the process of model formulation.

If we define $E_n$ as $E_n(t)=(k_{in}*f_n)(t)/{k_1}$, $n\ge 2$, then by differentiating $E_n$ we have the following system of ODEs as follows:

$$\begin{aligned} {\left\{ \begin{array}{ll} \frac{du}{dt}=k_{in}(u,w)-k_{out}(C,u)\\ \frac{dy}{dt}=k_{out}(C,u)-k_1E_n(t)\\ \frac{dE_n}{dt}=k_1(E_{n-1}(t)-E_n(t)), \end{array}\right. } \end{aligned}$$

provided with $E_i(0)=0$, $i=1,2,\ldots ,n$. If $y_i$ is the damaged tumor cells with age *i*, then the total damaged cells *y* is considered by $y=y_1+y_2+\cdots +y_n$. Let $y_i=E_i$. Then, the Erlang TCM was derived as

2 $$\begin{aligned} {\left\{ \begin{array}{ll} \frac{du}{dt}=k_{in}(u,w)-k_{out}(C,u)\\ \frac{dy_1}{dt}=k_{out}(C,u)-k_1 y_1(t)\\ \frac{dy_2}{dt}=k_1(y_1(t)-y_2(t))\\ \vdots \\ \frac{dy_n}{dt}=k_1(y_{n-1}(t)-y_n(t)), \end{array}\right. } \end{aligned}$$

satisfying ${dy}/{dt}={d}(y_1+y_2+\cdots +y_n)/{dt}=k_{out}(C,u)-k_1E_n$. The schematic diagram of the resulting Erlang TCM is shown in Fig. 1a.

### Derivation of Coxian TCM

Unlike Erlang TCM we derive above, TCM using Coxian distribution has the following systemic situation. Some of the damaged cells cascade multiple-step transitions with a chain of compartments and the others at age *i* are eliminated directly. This situation seems natural because all cells are not only systemically eliminated by the transition process, but also they happen sudden death by the change in the cell cycle^20^. To formulate the processes, we consider a phase-type distribution, which is the distribution of time-based on a continuous-time Markov process written in the form of a transition rate matrix, as follows:

$$\begin{aligned} Q= \begin{pmatrix} 0&{}0\\ S^0&{}S\\ \end{pmatrix}, \end{aligned}$$

where *S* is $n\times n$ matrix of a transition rate matrix. $S^0=-S\mathbf{1}$ and $\mathbf{1}$ represent an $n\times 1$ column vector, with every element being 1. Then, the distribution function of *t* is given by $F(t)=1-\alpha \cdot exp(St)$ and its density *f* as $f(t)=\alpha \cdot exp(St)S^0$, with probability row vector $\alpha$^21^. As a direct consequence, we have

3 $$\begin{aligned} \frac{df}{dt}=f\cdot S, \end{aligned}$$

where $f=(f_1,f_2,\ldots ,f_n)$. For example, if $\alpha =1$ and $S=-\lambda$, then *f* becomes the density of the exponential distribution. In addition, if $\alpha =(1,0,0,\ldots ,0)$ and *S* is an $n\times n$ matrix with diagonal entries $-k_1$ and superdiagonal entries $k_1$ with $f_1(0)=1$, then we derived the Erlang distribution. The Coxian distribution is a generalization of the Erlang distribution, as follows: $\alpha =(1,0,0,\ldots ,0)$ and *S* is an $n\times n$ matrix with diagonal entries $-k_i$, $i=1,2,\ldots ,n$ and superdiagonal entries $p_ik_i$, $i=1,2,\ldots ,n-1$, where $f_1(0)=1$. Since a problem of modeling using Coxian distribution is no analytic form of the density function, the direct approach using differentiation is unavailable, unlike Erlang distribution. Instead, they satisfy the following relations above, and the model approach is as follows. Let $f_i$, $i=1,2,\ldots ,n$ be the *i*th component of the Coxian density. By the consideration above, we have

$$\begin{aligned} \frac{df_1}{dt}=-k_1f_1,~\frac{df_i}{dt}=p_{i-1}k_{i-1}f_{i-1}-k_if_i, ~i=2,\ldots ,n. \end{aligned}$$

As shown in Eq. (1), we define the total damaged cells *y* and $k_{out}*f$ as $y=y_1+y_2+\cdots +y_n$ and $(k_{out}*f)(t)=\sum _{i=1}^n(k_{out}*f_i)(t)$, respectively. Herein, $y_i$’s are the number of damaged cells at age *i*. We assume $p_n=0$ and $(1-p_n)k_n=\cdots =(1-p_{i-1})k_{i-1}=\cdots =(1-p_1)k_1$. This assumption is necessary for the convolution form $k_{out}*f_i$ to induce a similar relation between density functions. Let $y_i=({k_{out}*f_i})/{[(1-p_i)\cdot k_i]},~i=1,2,\ldots , n$. Then the rate of change $y_i$ can be calculated and given by

$$\begin{aligned} \frac{dy_i}{dt}=p_{i-1}k_{i-1}y_{i-1}\cdot \frac{(1-p_{i-1})k_{i-1}}{(1-p_i)k_i}-k_iy_i=p_{i-1}k_{i-1}y_{i-1}-k_iy_i. \end{aligned}$$

These considerations give rise to the following system of ODEs:

4 $$\begin{aligned} \begin{bmatrix} \frac{dy_1}{dt}\\ \frac{dy_2}{dt}\\ \vdots \\ \frac{dy_{n}}{dt}\\ \end{bmatrix}= \begin{bmatrix} k_{out}(C,u)\\ 0\\ \vdots \\ 0\\ \end{bmatrix}+ \begin{bmatrix} -k_1y_1\\ p_1k_1y_1-k_2 y_2\\ \vdots \\ p_{n-1}k_{n-1}y_{n-1}-k_{n}y_{n}\\ \end{bmatrix}. \end{aligned}$$

In addition, this approach satisfies

$$\begin{aligned} \frac{d(y_1+y_2+\cdots +y_n)}{dt}=k_{out}(C,u)-\sum _{i=1}^n(1-p_i)k_iy_i=k_{out}(C,u)-\sum _{i=1}^n(k_{out}*f_i)(t). \end{aligned}$$

To further simplify the model, we substitute the average values in place of $p_i$ and $k_i$, that is, $p=\frac{1}{n}\sum _{i=1}^np_i$ and $k_1=\frac{1}{n}\sum _{i=1}^nk_i$. Then these approaches satisfy the assumptions. Substituting them into Eq. (4), associated with the first equation in Eq. (2). Then, Eq. (1) gave rise to the following system of ODEs:

5 $$\begin{aligned} {\left\{ \begin{array}{ll} \frac{du}{dt}=k_{in}(u,w)-k_{out}(C,u)\\ \frac{dy_1}{dt}=k_{out}(C,u)-k_1 y_1,\\ \frac{dy_2}{dt}=pk_1y_1-k_1y_{2},\\ ~~~~~~~~~~~ \vdots \\ \frac{dy_{n}}{dt}=pk_1y_{n-1}-k_1y_{n},\\ \end{array}\right. } \end{aligned}$$

which considers the death probability of some cells during one another transition. The schematic diagram of the resulting Coxian TCM is shown in Fig. 1b.

### Growth and mortality functions

Simeoni and colleagues developed a well-known perturbed growth (tumor) model, which is a special type of Erlang TCM^2^. We call this TCM as Simeoni TCM. Their model was applied to describe the delays for capturing tumor data. They presented the growth function $k_{in}$ and mortality function $k_{out}$ given by

$$\begin{aligned} k_{in}(u,w)=\frac{\lambda _0 u}{\big (1+\big (\frac{\lambda _0}{\lambda _1}w\big )^\phi \big )^{\frac{1}{\phi }}}~ \mathrm{and}~ k_{out}(C,u)=\eta \cdot C\cdot u. \end{aligned}$$

Note that for a sufficiently large $\phi$ (more than 10), $k_{in}$ is approximately exponential growth with a linear rate $\lambda _0 u$ less than or equal to the threshold $w_{th}={\lambda _1}/{\lambda _0}$ and non-linear growth with a rate of $(\lambda _1\cdot u)/{w}$ otherwise. They described two growth rates in a single form for computational reasons. Also, they considered the number of the compartments of the damaged cells as four to capture the delay process.

### Pharmacokinetic model

The pharmacokinetic (PK) model was from the Magni study and was also applied to Simeoni TCM^7^. PK model consists of two compartments as follows:

6 $$\begin{aligned} {\left\{ \begin{array}{ll} \frac{dq_1}{dt} =-k_{01}q_1(t)-k_{21}q_1(t)+k_{12}q_2(t)+v(t)\\ \frac{dq_2}{dt} =k_{21}q_1(t)-k_{12}q_2(t)\\ C(t)=\frac{q_1(t)}{V}, \end{array}\right. } \end{aligned}$$

where $q_1$ and $q_2$ are the amounts of drug in the plasma and peripheral compartments, respectively, and *V* is the volume in plasma. $C(t) (ng\cdot ml^{-1})$ is the concentration, and *v*(*t*) is the bolus administration ($ng\cdot kg^{-1}$). Such obtained drug concentration *C* is applied to Eqs. (2) and (5) for the comparison between Simeoni and Coxian TCMs with $n=4$.

### Parameter values

**Figure 2:**
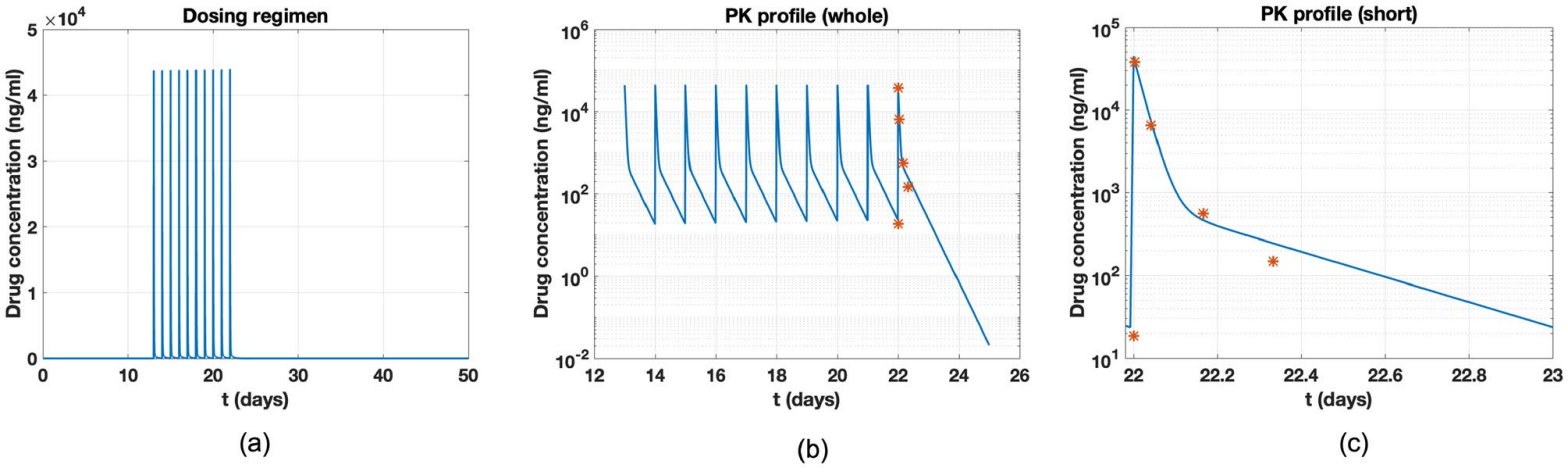
(**a**) 10 times dosing regimen per day from day 13 is plotted. (**b**) PK profile are plotted. Red one represents observed data after final injection. (**c**) PK profile in the study^7^ is reproduced.

Parameter estimations and model simulations are performed in both TCMs based on the data set. Data set consists of tumor size vs. time profile, as shown in *mouse 150*^7^. The tumor was implanted on day 0 with an initial size of 0.0121*g*, and the drug was administered on day 13 and injected every day for 10 days. We reproduce their study and plot PK profile detail, as shown in Fig. 2. The parameter values are the same except for *p*: For the PK model, $k_{01}=1.6$, $k_{21}=0.2353$, $k_{12}=0.1699$ and $V=1028$ and for per injection time $t_{in}$, $q_1(t_{in})=4.5\times 10^7$. $t_{in}$ represents dosing timing for 10 injections. For the tumor model, initial tumor size $w_0=0.0121$, $\lambda _0=0.25$, $\lambda _1=0.4603$, $\phi =20$, $\eta =0.7816$ and $k_1=0.2859$. All simulations were conducted by Matlab 2021a using the ODE45, ODE solver based on Runge-Kutta method and lsqnonlin function, a nonlinear optimization for parameter estimation. The only difference between both models is *p* and the estimated value is 0.44268.

**Figure 3:**
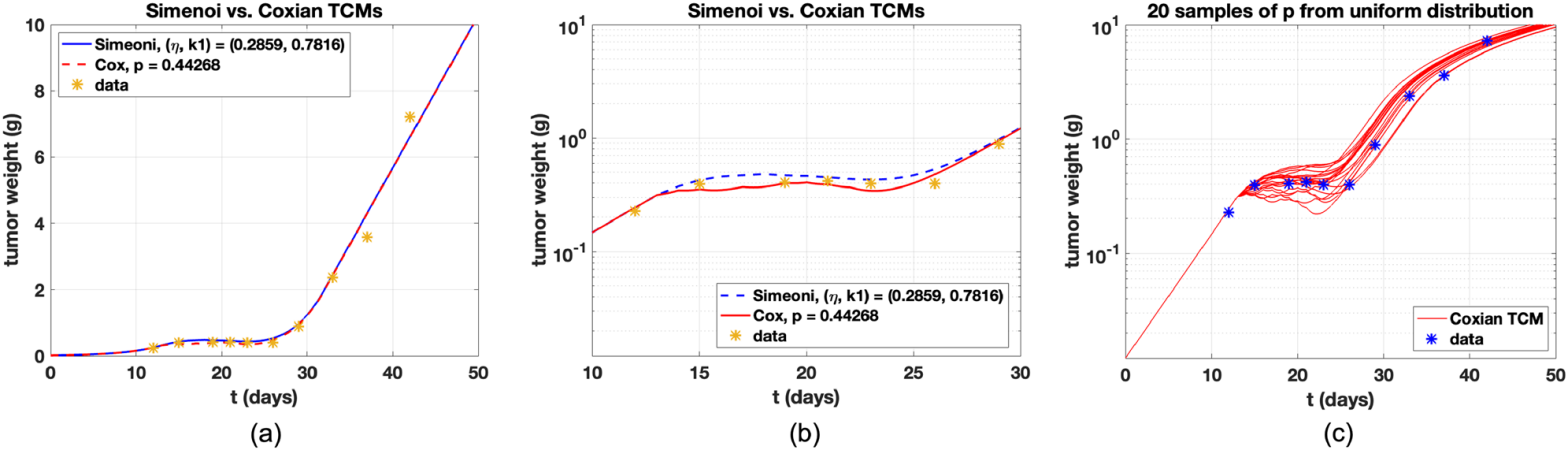
Simeoni and Coxian TCMs are compared. The number of the damaged cells is $n=4$ and the total number of the cell compartments is five in both TCMs. All parameters are the same. (**a**) Both dynamics look similar in the linear scale. (**b**) Log-scale is applied and time is limited from day 10 to day 30. Dynamics conducted by Coxian TCM show less delay in the drug effect phase. (**c**) Twenty samples of *p* are collected and dynamics are plotted. Some of dynamics show stronger delay.

### Comparison between Simeoni and Coxian TCMs

**Figure 4:**
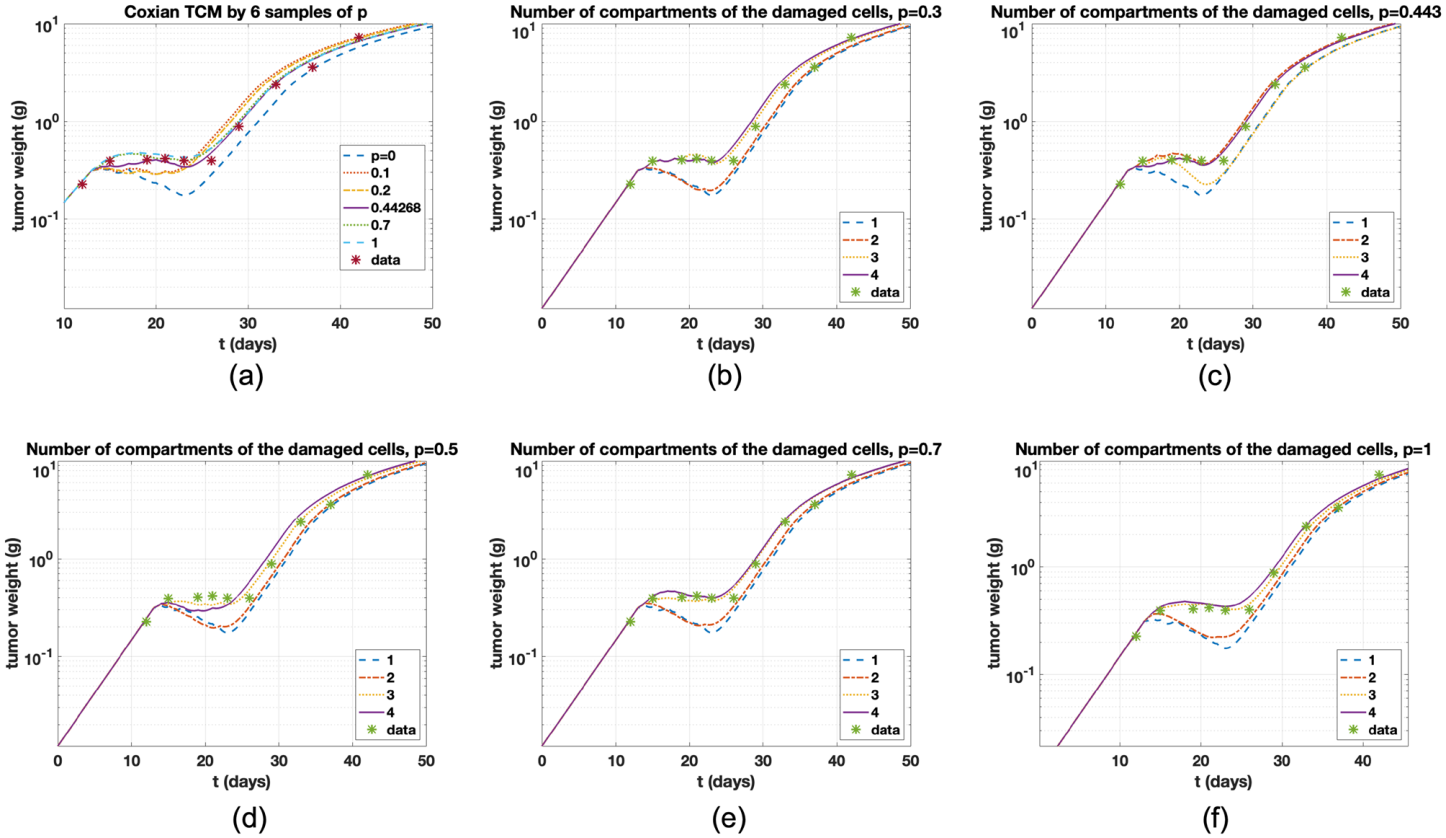
(**a**) For the change of delay, dynamics of Coxian TCM are investigated by various *p*’s. The number of the compartments of the damaged cells is $n=4$. $p=1$ represents Erlang TCM. (**b**)–(**e**) Delays are differently determined by the number of the compartments depending on *p*. Delay is not followed as regular sequences. (**f**) In Erlang TCM, tumor delay follows the regular sequence.

PK Profile using Eq. (6) is shown in Fig. 2. All parameters are from the study^7^. The predicted curve captures the bi-exponential behavior that describes the typical PK of anticancer therapy. Simeoni and Coxian TCMs with the number of the compartments of the damaged cells, $n=4$, are compared shown in Fig. 3a. Their dynamics look similar, but we change the y-axis as log-scale and time axis to see the difference, as shown in Fig. 3b. After day 13 that is the first injection, inhibition of the tumor cells is seen, and the drug effect phase is considered around day 13 to 30. We measure normalized root mean square error (n-RMSE). n-RMSE is given by $\sqrt{{\sum _{i=1}^n (y_i-\hat{y_i})^2}/{n}}/(y_{max}-y_{min})$. $y_i$ is the *i*th observation and $\hat{y_i}$ is the corresponding value given the model. Also, the number of the data is $n=10$, and $y_{max}$ and $y_{min}$ are the maximum and minimum values given measured data. Measured n-RMSEs for Erlang and Coxian TCMs are 0.1062 and 0.0745, respectively, showing that Coxian TCM fits data more reliable than Simeoni TCM. To explore the influence of *p*, twenty samples of *p* are chosen and plotted in Fig. 3c. If $p=1$, then Coxian TCM is exactly Simeoni TCM. Notably, some dynamics are over that of Simeoni TCM, indicating stronger delays. The various values of *p* are investigated and plotted for the change of delays in Fig. 4a. $p=0$ shows the biggest inhibition in tumor weight during the drug effect period. Our expectation is that if *p* increases, then delays are stronger, but not likely from the simulation results. The change of *p* does not assure in regular sequence in multiple dosing regimens. We investigate the effect of *p* depending on the number of the compartment of damaged cells, *n*. In Fig. 4, the values of *p* are differently given, and the change of *n* is explored in the figures. In Simeoni TCM, *n* determines the magnitude of the tumor delay. That is, if *n* is increasing, then the delay is stronger shown in Fig. 4f. However, in the Coxian model, *p* also affects the change of the delay but not follows regularly as *n* shown in Fig. 4b–e. Rather the change of *p* captures various delays. Thus, the sophisticated delays can be expressed by tuning *p*. Consequently, the change of *p* may induce heterogeneous dynamical changes of delays in multiple dosing regimens, as shown in Fig. 4. Here, heterogeneous dynamics mean that their delay effect is not followed by regular sequences. This phenomenon is also seen in Fig. 4a,d. The Coxian model approach, combined with the number of the compartments and variation of *p*, enables more diverse tumor inhibition cases upon different dosing regimens.

**Figure 5:**
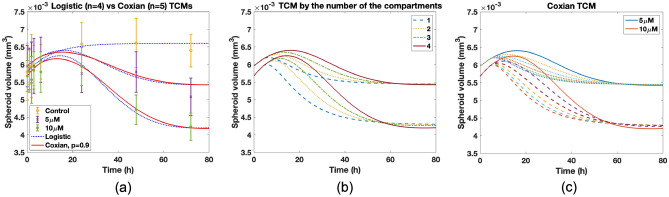
(**a**) Data fit using Erlang and Coxian TCMs with logistic growth. Parameters are estimated for three data set using Erlang TCM with $n=4$. In Coxian TCM with $n=5$, all parameters are the same as Erlang TCM and $p=0.9$. (**b**) In logistic TCM, dynamics are presented by the change of the number of the compartments. Control case is excluded. (**c**) In Coxian TCM with $n=4$, *p* is variated from 0 to 1 with interval 0.2 and delays are compared to (**b**).

We explore the differences between *p* and the number of compartments for a single injection. In this case, lapatinib data is used to clarify the relationship. Single drug concentration with control, 5 µM and 10 µM was injected into the tumor cells. We measured the tumor size at specific times after injection. The detailed process of in vitro experiment is shown in “Materials” section. Modeling is provided with the growth function whose form is given by a logistic growth $k_{in}=\lambda _0\cdot (1-w/u_{max})u, w=u+y_1 +y_2+\cdots +y_n,$ since the final tumor size is limited. The mortality function and others are the same. We call Erlang TCM with the logistic growth as logistic TCM. The number of the damaged compartments, *n*, is four in logistic TCM and five in Coxian TCM. Drug concentration does not change as time elapses because short time and no elimination process is considered. From control data using logistic TCM, $\lambda _0$ and $u_{max}$ are estimated by 0.12 and 0.0066. Using two data sets of 5 µM and 10 µM, $k_1$ and $\eta$ are estimated by 0.1682 and 0.0035. In Coxian TCM, all parameters are the same, and *p* is estimated by 0.9. Data fit with data variance is shown in Fig. 5a. We measure n-RMSE for each case (control, 5 µM and 10 µM). Also, the number of the data per the case is eight. For control case, n-RMSE is 0.1032. For 5 µM, 0.2385 and 0.227 for logistic and Coxian TCM, respectively. For 10 µM, 0.0815 and 0.0774 in turn, showing that fit quality of Coxian TCM is better. We compare the change of *p* and the number of the compartments. Fixing $n=4$ in both models, *p* is variated from zero to one with interval 0.2. In Erlang TCM, the number of the compartment is changed. Delays positively follow the number of the compartments and the increase in *p*, showing that magnitude of delay seems similar, but the change of *p* enables various dynamics, as shown in Fig. 5b,c. This result shows that variation of *p* enables more delicate delay expression than the change of the number of the compartments. Coxian TCM reflects the realistic situation considering the sudden death of the cells during the transit processes with the help of *p* and enables that the cells of the same age do not have the same death. Consequently, Coxian TCM is the extended model that can capture various delays and provide realistic death processes of the damaged cells.

### Equilibrium upon constant drug infusion

**Figure 6:**
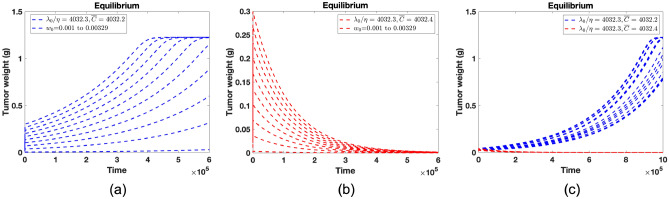
(**a**) $\lambda _0/\eta$ is 4032.3 which represents threshold. Constant drug infusions are given by 4032.2. $w_0$’s are variated from 0.001 to 0.00329 with the constant intervals. Drug infusion is 4032.2. Tumor has non-zero equilibrium. (**b**) Drug infusion rate is 4032.4 with the same initial tumor size in (**b**). Tumor has zero equilibrium. (**c**) Initial tumor size $w_0$ is fixed by 0.0121. The equilibrium points are changed according to the value of *p*. Ten points of *p* are chosen from uniform distribution [0, 1]. The equilibrium points are decreasing as *p* is increasing when $\overline{C}$ is less than $\lambda _0/\eta$.

We investigate the change of the equilibrium according to constant drug infusion. If the drug is infused, given by $\overline{C}$, the slight change of infusion $\overline{C}$ triggers the difference in equilibrium, as shown in Fig. 6. This depends on the ratio $\lambda _0/\eta$, and does not change in variations of initial tumor sizes shown in Fig. 6a,b. Equilibrium depends on the value of *p* shown in Fig. 6c. Non-zero equilibrium is decreasing as *p* is increasing when $\overline{C}$ is less than $\lambda _0/\eta$. We also present the steady-state analysis at equilibrium points mathematically. Proofs are shown in Supplementary Information.

#### **Proposition 1**

*Let* $k_{in}(u,w)={\lambda _0 u}/{\big (1+\big (\frac{\lambda _0}{\lambda _1}w\big )^\phi \big )^{\frac{1}{\phi }}}~ \mathrm{and}~ k_{out}(C,u)=\eta \cdot C\cdot u$ *from* Eq. (5). *If* *drug* *C*(*t*) *is administered through an infusion, yielding a (steady state) constant concentration* $C=\overline{C}$. *If* (i) $\overline{C}>{\lambda _0}/{\eta },$ *then the only equilibrium point is* $\overline{u}=\overline{y_1}=\cdots =\overline{y_n}=0$. (ii) $\overline{C}<{\lambda _0}/{\eta }$, *then two equilibrium points are* $\overline{u}=\overline{y_1}=\cdots =\overline{y_n}=0$ *and* $\overline{u}=\frac{\lambda _1}{\eta \overline{C}+\frac{(\eta \overline{C})^2}{k_1}\cdot \frac{1-p^{n}}{1-p}}$, $\overline{y_1}=\eta \overline{C}\frac{\overline{u}}{k_1}$, $\ldots$, $\overline{y_{i}}=\frac{\eta \overline{C}\overline{u}}{k_1}p^{i-1}$, $i=1,2,\ldots ,n$, *and*

$$\begin{aligned} \overline{w}={\left\{ \begin{array}{ll} \overline{u}+\sum _{i=1}^{n}\overline{y_i} =\big (1+\frac{\eta }{k_1}{\overline{C}}\cdot \frac{1-p^{n}}{1-p}\big )\overline{u}, ~if~ p<1\\ \big (1+n\overline{C} \frac{\eta }{k_1}\big )\overline{u},~ if~ p=1. \end{array}\right. } \end{aligned}$$

(iii) $\overline{C}={\lambda _0}/{\eta }$, *then the infinite equilibrium points are* $\overline{u}=\frac{\overline{w}}{1+\frac{\eta }{k_1}\overline{C}\cdot \frac{1-p^{n}}{1-p}}\le \frac{\lambda _1}{\lambda _0+\frac{\eta }{k_1}\overline{C}\lambda _0\cdot \frac{1-p^{n}}{1-p}}$.

#### **Corollary 1**

*If* $k_{in}=\lambda _0\big (1-\frac{u}{w_{max}}\big )u$ *and* $k_{out}$ *are the same, then if* (i) $\overline{C}\ge \frac{\lambda _0}{\eta }$, *then there is a unique equilibrium zero*; (ii) $\overline{C}<\frac{\lambda _0}{\eta }$, then $\overline{u}=w_{max}\frac{\lambda _0-\eta \overline{C}}{\lambda _0}$, $y_i=\frac{\eta }{k_1}\overline{C} \overline{u} p^{i-1}$, $i=1,2,\ldots ,n-1$, $y_n=\frac{\eta }{k_1}\overline{C}\overline{u}$, and $w=\big (1+\frac{\eta }{k_1}\overline{C}\big (1+\frac{1-p^{n-1}}{1-p}\big )\big )u$.

#### **Proposition 2**

*Let* $k_{in}$ *be* *Simeoni*, *or the logistic growth rate. Then, the equilibrium point* $\overline{u}=\overline{y_1}=\cdots =\overline{y_n}=0$ *is unstable if* $\overline{C}<\frac{\lambda _0}{\eta }$ *and globally asymptotically stable if* $\overline{C}>\frac{\lambda _0}{\eta }$.

#### **Proposition 3**

*Let* $k_{in}$ *be* *Simeoni*, *or the logistic growth rate. If* $\overline{C}<\frac{\lambda _0}{\eta }$, *then the non-zero equilibrium points are asymptotically stable for* $n=2,3,4$.

## Discussion

The responses of cell populations have attracted attention in PKPD. TCMs describe delays or aging processes in the cell population after drug administration. In Erlang TCM, all damaged cells are eliminated through transit processes, and a system of ODEs expresses the processes. We develop the generalized TCM using an age-structured model, and a stochastic process using a survival function is applied to the transit process as a convolution of the distribution and degradation rate. Thus, we provide two delayed cell death processes by Erlang and Coxian distributions. From Erlang distribution, existing TCMs are derived. Another is Coxian TCM. This model considers that some of the cohorts could fall into sudden death. This model covered Erlang TCM and captured various delay processes using the probability *p*. The relation between *p* and the number of the compartments is compared in terms of delays in dynamics. Coxian TCM may reflect the flexible delay effect of the drug at the effect phase. In addition, equilibrium analysis is conducted to capture Coxian TCM’s characteristics determined by *p*. *p* affects the change in the nonzero equilibrium points.

One assumption was used for the Coxian model in the model establishment. $(1-p_1)k_1=\cdots =(1-p_{n-1})k_n$ means that the instant death rate is the same in all compartments. If this assumption does not hold, the model becomes more complicated as the number of the compartments increases. This disadvantage is shown in phase-type distribution except for Erlang distribution. To overcome this shortcoming, non-Markovian distribution such as Mittag-Leffler (ML) distribution can be applied^22^. There is no explicit closed-form of density function *f* or a differential equation to interact with the relationship in the ML distribution. However, fractional derivatives can be derived by using the appropriate Laplace transformation. Finding the biological significance of this model is the subject of the next study.

Prediction after the model formulation is considered. Once the number of compartments is determined in the TCM model, parameters can be estimated for the data. Here, two problems may arise. The first is the prediction of a new dosing regimen after a dosing regimen, and the second is the dynamics when the drug concentration is changed. It is a problem of determining the number of compartments in TCMs. The compartments can be chosen to reflect the complex processes within the cells of the drug. For example, in the case of an antibody-drug conjugate, it undergoes a receptor-mediated endocytosis process after binding to a target antigen. The drug has a mechanism of inhibiting cancer through early endosome, late endosome, and lysosomal degradation processes, etc^23,24^. At least three compartments are required. Determination of the number of compartments is biologically important, but in most cases, it is used to fit responses such as changes of the cells. From the model point of view, the Coxian model somewhat compensates for this shortcoming by using *p*. We conducted the study using the average value *p* instead of $p_i$, but it seems that it will be possible to generate results if we use $p_i$ after determining the number of biological compartments. The second point is predicting dynamics when changing the dose concentration. In an experiment using lapatinib, both models did not fit well when predictions were conducted for 20 µM after using estimated parameters that fit the data using the concentration of control, 5, 10 µM. For this reason, the drug effect is saturated and does not change above some concentrations. To reflect this, the drug effect model can be applied to TCM. However, this study focused on studying the diversification of the delay effect using *p*. Despite the advantage of TCM using Coxian distribution, determining all $p_i$ values is challenging for estimating values due to the number of parameters depending on the transit compartments. In this study, this difficulty is resolved by the mean value *p* of $p_i$, but this approach loses the nature of each transit compartment and only reflects the indirect elimination process directly. Each transit compartment may have a different MRT in a real situation, but it is unlikely to measure all experimentally. However, inferring some $p_i$’s could be valuable in the essential transit processes.

Several Erlang TCMs with various growth and mortality functions are widely used in PKPD^2,7,10^, epidemics^25,26^, and other research areas^27–29^. In PKPD, they measured tumor growth and the effect of anticancer treatment using the TCMs, associated with parameter estimation of data. In epidemics, Erlang TCMs are used for determining latent and infectious periods or vaccination periods. Also, Erlang TCM is applied to measure time elapse for cell infection and virus production, describe the interactions between the immune system and tumor cells taking into account distributed time delays, or compare population models with delayed continuous-time Markov chains. Using Erlang distribution, they obtain a simplistic result regarding time delays. However, these approaches could miss realistic time delays in the phase^30^. Thus, we believe that TCM obtained by Coxian distribution could be an alternative to compensate for the limitation.

## Materials and methods

### Materials

A lapatinib-free base was purchased from LC Laboratories (Woburn, MA, USA). RPMI 1640 (containing 300 mg/L of L-glutamine, 25 mM HEPES, and 25 mM NaHCO3) cell culture medium, penicillin-streptomycin, and fetal bovine serum (FBS) were obtained from Gibco Life Technologies, Inc. (Carlsbad, CA, USA). All other reagents were of analytical grade and were purchased from commercial sources.

### Incubation time and cell seeding density-dependent proliferation assay

Incubation time- and cell seeding density-dependent cell proliferation profiles were tested in SK-BR-3 (human breast adenocarcinoma) cells using an MTS-based assay^31,32^. SK-BR-3 cells were purchased from the Korean Cell Line Bank (Seoul, Korea). A mixture of RPMI 1640, heat-inactivated FBS (10%, v/v), and penicillin-streptomycin (1%, v/v) was used as the complete cell culture medium for SK-BR-3 cells. SK-BR-3 cells were seeded onto 96-well plates (at a density of $2.0 \cdot 103$, $5.0 \cdot 103$, and $1.0 \cdot 104$ cells per well) and incubated for 0, 24, 48, and 72 *h* at $37^{\circ }\hbox {C}$ ($n = 5$ in each group). Cells were treated with CellTiter $96^{\circledR }$ AQueous One Solution Cell Proliferation Assay Reagent (Promega Corp., Fitchburg, WI, USA) and processed according to the manufacturer’s protocol^31^. After incubating for 1 *h* at $37^{\circ }\hbox {C}$, the absorbance values at 490 nm were estimated using a plate reading spectrophotometer (SpectraMax i3; Molecular Devices, Sunnyvale, CA, USA)^33^.

### Anticancer activity test in 3D spheroid model

The spheroid growth inhibition study of lapatinib was assessed using a 3D spheroid model of SK-BR-3 cells. SK-BR-3 cells were cultured in an AggreWellTM400 24-well plate (STEMCELL Technologies Inc., Vancouver, BC, Canada). Cells ($2.4 \cdot 105$ cells) prepared in RPMI 1640 (containing 300 *mg*/*L* of L-glutamine, 25 *mM* HEPES, and 25 *mM* NaHCO3), including heat-inactivated FBS (10%, v/v), and penicillin-streptomycin (1%, v/v), were applied to each well and spheroids were formed according to the manufacturer’s directions^34^. The cell culture media were gently replaced every other day for 96 h. The spheroids were treated with lapatinib (5, 10, and 20 µM) and incubated at $37^{\circ }\hbox {C}$ in complete cell culture medium ($n = 30$ in each group). After cultivation for 0, 1, 2, 3, 6, 24, 48, and 72 h, the shape of SK-BR-3 cell spheroids was observed by inverted microscopy (Eclipse TS100-F; Nikon, Tokyo, Japan). The volume (*V*, $mm^3$) of the spheroid was calculated using the following formula: $0.5\cdot (long~ diameter) \cdot (short~ diameter)^2$^35^.

### Methods

#### Mathematical formulation: age-structured based perturbed tumor model using survival function

Consider a cohort of cells, a group of cells of age in an interval of length $\Delta a$. Then, we obtain the Mckendrick-von Foerster equation as follows:

7 $$\begin{aligned} {\left\{ \begin{array}{ll} \frac{du}{dt} = k_{in}(u,w)-k_{out}(C,u)\\ \frac{\partial \phi }{\partial t}+\frac{\partial \phi }{\partial a}\cdot \frac{da}{dt}=-\mu (a,C)\phi (a,t), \end{array}\right. } \end{aligned}$$

where $u=u(t)$ is the number of proliferating cells with initial $u(0)=u_0$ and $C=C(t)$ is the drug concentration. The total number of damaged cells at time *t* is given by $y(t)=\int _0^\infty \phi (a,t)da$. $w=w(t)$ is the total number of cells given by $w(t)=u(t)+y(t)$. We expect that the unit of age is the same as that of time and so assume ${da}/{dt}=1$. In addition, if the mortality rate $\mu (a,C)$ depends only on age *a*, then $\mu (a,C)=\mu (a)$. This assumption is reliable because the age depends on the duration after the drug *C* is injected. At $a=0$, the boundary condition is $\phi (0,t)=k_{out}(C,u)$, meaning that damaged tumor cells of age zero are created by tumor size and drug concentration. The initial condition of $\phi$ is given by $\phi (a,0)=0$, indicating that all tumors without drug administration are proliferating cells. Under these conditions, time *t* represents the time since administration of the drug.

By the method of characteristics, the existence and uniqueness of the solution for Eq. (7) was proved, which can be solved as follows^36^:

8 $$\begin{aligned} \phi (a,t)= {\left\{ \begin{array}{ll} k_{out}(C(t-a),u(t-a))e^{\int _0^\infty \mu (\alpha )d\alpha },~~t\ge a\\ 0,~~t<a, \end{array}\right. } \end{aligned}$$

which holds that $\phi (a,t)=k_{out}(C(t-a),u(t-a))e^{\int _0^\infty \mu (\alpha )d\alpha }$ for all $t\ge 0$. In addition, we integrated the second equation in Eq. (7) over the age *a*. Then

9 $$\begin{aligned} \frac{dy}{dt}=k_{out}(C,u)-\int _0^\infty \mu (a)\phi (a,t)da. \end{aligned}$$

The mortality rate $\mu (a)$ is related to the probability of survival. To see this, we consider a stochastic process. Let $S(a)={{Pr}}\{T\ge a\}$ be the probability of survival from zero to age *a*. Then, *S*(*a*)*y* denotes the number of cells from the cohort with age *a*, and the number of cells that die in $\Delta a$ is given by $S(a+\Delta a)y-S(a)y=-\mu (a)S(a)y\Delta a$. Dividing both sides by $y\Delta a$ and $\Delta a\rightarrow 0$. Then,

$$\begin{aligned} \frac{dS}{dt}=-\mu (a)S(a). \end{aligned}$$

Because the initial value of *S* was the probability of survival until age 0, we assumed $S(0)=1$. Solving this equation, we found $S(a)=exp({-\int _0^\infty \mu (\alpha )d\alpha })$, whose derivative defined the probability density function *f* of ${{Pr}}\{T<a\}$, such that $f(a)=-{dS}/{da}=\mu (a)S(a)$. This indicates the probability that a cohort dies within age *a*. Thus, $\mu$ can be interpreted as the death hazard rate. Substituting Eq. (8) into Eq. (9) together with *f*, we derived the following equation:

10 $$\begin{aligned} \frac{dy}{dt}&= k_{out}(C,u)-\int _0^\infty k_{out}(C(t-a),u(t-a))f(a)da\nonumber \\&= k_{out}(C,u)-(k_{out}*f)(t), \end{aligned}$$

where $*$ denotes the convolution operator. Thus, we obtained the master equation for the perturbed tumor model as follows:

11 $$\begin{aligned} {\left\{ \begin{array}{ll} \frac{du}{dt}=k_{in}(u,w)-k_{out}(C,u)\\ \frac{dy}{dt}=k_{out}(C,u)-(k_{out}*f)(t), \end{array}\right. } \end{aligned}$$

associated with $u(0)=u_0$, $y(t)=\int _0^\infty \phi (a,t)dt$, and $w(t)=u(t)+y(t)$. The mortality (elimination) rate of collection of damaged cells *y* was delayed and given in the form of convolution.

## Supplementary Information

Supplementary Information 1.

Supplementary Information 2.

## Data Availability

All data generated or analysed during this study are included in this published article and its supplementary information files.
