# Supplementary material for: Extended transit compartment model to describe tumor delay using Coxian distribution
Source: Sci Rep. 2022 Jun 16;12:10086. doi: 10.1038/s41598-022-13836-4 (PMC9203540; doi:10.1038/s41598-022-13836-4)
Supplement: Supplementary file 1 — Supplementary Information 1. [file 41598_2022_13836_MOESM1_ESM.pdf]

Extended transit compartment model to describe tumor delay using Coxian distribution Supplementary Information

We restrict our interest probability to $0 \leq p < 1$. For $p = 1$, it has already been proven in the study[1].

Proof of *Proposition* 1. From Eq.(5) associated with $k_{in}(u,w) = \lambda_0 u/\big(1+\big(\frac{\lambda_0}{\lambda_1}w\big)^{\phi}\big)^{\frac{1}{\phi}}$ and $k_{out}(C,u) = \eta \cdot C \cdot u$, let $\frac{du}{dt} = \frac{dy_1}{dt} = \cdots = \frac{dy_n}{dt} = 0$. Then $\bar{y_1} = \frac{k_2}{k_1}\bar{C}\bar{u}, \cdots, y_n = \frac{k_2}{k_1}\overline{C}\bar{u}p^{n-1}$, where $\bar{u}$ is the equilibrium point of u. Because $\overline{w} = \overline{u} + \sum_{i=1}^{n}\overline{y_i}$,

$$\overline{w} = \Big(1+\frac{k_2}{k_1}\overline{C}\cdot\frac{1-p^n}{1-p}\Big)\overline{u}. \tag{1}$$

Case I. If $\overline{w} \leq \frac{\lambda_1}{\lambda_1}$, then $k_{in}(\overline{u},\overline{w}) = \lambda_0\overline{u}$, so $\lambda_0\overline{u} - k_2\overline{C}\overline{u} = 0$. If $\overline{C} \neq \frac{\lambda_0}{k_2}$, then $\overline{u} = 0$ and $\overline{w} = 0$. If $\overline{C} = \frac{\lambda_0}{k_2}$, then for every $\overline{u}$, $\overline{w}(\leq \frac{\lambda_1}{\lambda_0})$ is an equilibrium point. By Eq. (1),

$$\overline{u} = \frac{\overline{w}}{1+\frac{k_2}{k_1}\overline{C}\cdot\frac{1-p^n}{1-p}} \leq \frac{\lambda_1}{\lambda_0+\frac{k_2}{k_1}\overline{C}\lambda_0\cdot\frac{1-p^n}{1-p}}.$$

Case II. If $\overline{w} > \frac{\lambda_1}{\lambda_0}$, then $k_{in}(u,w) = \lambda_1\frac{u}{w}$. Then, $0 = \frac{du}{dt} = \lambda_1\frac{\overline{u}}{\overline{w}} - k_2\overline{C}\overline{u}$. Because $\overline{u} \neq 0$, $\overline{w} = \frac{\lambda_1}{k_2\overline{C}} < \frac{\lambda_1}{\lambda_0}$. This shows that $\overline{C} < \frac{\lambda_0}{k_2}$. Therefore, using Eq. (1) again,

$$\overline{u} = \frac{\overline{w}}{1+\frac{k_2}{k_1}\overline{C}\cdot\frac{1-p^n}{1-p}} = \frac{\lambda_1}{k_2\overline{C}+\frac{(k_2\overline{C})^2}{k_1}\cdot\frac{1-p^{n-1}}{1-p}}.$$

Proof of *Corollary* 1. Let $\frac{du}{dt} = \frac{dy_1}{dt} = \cdots = \frac{dy_n}{dt} = 0$.Then, $y_i = \frac{k_2}{k_1}\overline{C}\overline{u}p^{i-1}$, $i = 1,2,\cdots,n$, and $\overline{w} = \Big(1+\frac{k_2}{k_1}\overline{C}\frac{1-p^n}{1-p}\Big)\overline{u}$. Let $0 = \frac{du}{dt} = \lambda_0\big(1-\frac{\overline{u}}{w_{max}}\big) - k_2\overline{C}\overline{u} = 0$. Then, $\overline{u} = 0$ or $\overline{u} = w_{max}\frac{\lambda_0 - k_2\overline{C}}{\lambda_0}$.

Case I. If $\overline{C} \geq \frac{\lambda_0}{k_2}$, then there is a unique equilibrium $\overline{u} = 0$ and $\overline{y_i} = 0$ for all $i = 1,2,\cdots,n$.

Case II. If $\overline{C} < \frac{\lambda_0}{k_2}$, then $\overline{u} = w_{max}\frac{\lambda_0-k_2\overline{C}}{\lambda_0}$ and $\overline{w} = \Big(1+\frac{k_2}{k_1}\overline{C}\cdot\frac{1-p^n}{1-p}\Big)w_{max}\frac{\lambda_0-k_2\overline{C}}{\lambda_0}$.

Proof of *Proposition* 2. If $\overline{w} \leq \frac{\lambda_0}{\lambda_1}$, then $k_{in}(u,w) = \lambda_0 u$. Then, the associated characteristic polynomial is $\Psi(\rho) = det(\rho I - A)$, where the matrix A is given by

$$A = \begin{pmatrix} \lambda_0 - k_2\overline{C} & 0 & \cdots & 0 \\ k_2\overline{C} & -k_1 & \cdots & 0 \\ 0 & pk_1 & \cdots & 0 \\ \vdots & \vdots & \ddots & \\ 0 & 0 & \cdots & -k_1 \end{pmatrix}_{(n+1)\times(n+1)}.$$

Then, $\Psi(\rho) = (\rho - \lambda_0 + k_2\overline{C})(\rho + k_1)^n$. If $\overline{C} < \frac{\lambda_0}{k_2}$, then there is a real positive root, and thus, the equilibrium point is unstable. Conversely, if $\overline{C} > \frac{\lambda_0}{k_2}$, then all roots have negative real roots, and the equilibrium point is asymptotically stable. To show global stability, for $\overline{C} > \frac{\lambda_0}{k_2}$ and $w \leq \frac{\lambda_1}{\lambda_0}$ with $u(0) > 0$, $\frac{du}{dt} = \lambda_0 u - k_2 C u < \lambda_0 u - \lambda_0 u = 0$. In addition, if $\overline{C} > \frac{\lambda_0}{k_2}$ and $w > \frac{\lambda_1}{\lambda_0}$, then $\frac{du}{dt} = \frac{\lambda_1 u}{w} - k_2 cu = (\frac{\lambda_1}{w} - k_2 c)u < (\frac{\lambda_1}{w} - \lambda_0)u < (\lambda_0 - \lambda_0)u = 0$. This holds for $u > 0$ and $\frac{du}{dt} < 0$ for all t. Therefore, $u \to \hat{u} = 0$ as $t \to \infty$. The case of logistic growth is similar to that of the Simeoni growth.

Proof of *Proposition* 3. If $\overline{w} > \frac{\lambda_1}{\lambda_0}$, then $k_{in}(u,w) = \lambda_1\frac{u}{w}$. To study the local stability, we linearize the nonlinear system at the equilibrium point and analyze the stability of the linearized system. The system is as follows:

$$\begin{cases} \frac{du}{dt} = -\frac{\overline{u}\lambda_1}{\overline{w}^2}u - \frac{\overline{u}\lambda_1}{\overline{w}^2}y_1 - \cdots - \frac{\overline{u}\lambda_1}{\overline{w}^2}y_n \\ \frac{dy_1}{dt} = \overline{C}k_2 u - k_1 y_1 \\ \vdots \\ \frac{dy_{n-1}}{dt} = pk_1 y_{n-2} - k_1 y_{n-1} \\ \frac{dy_n}{dt} = pk_1 y_{n-1} - k_1 y_n. \end{cases}$$

The first line of the linearized system is achieved by letting $u-\overline{u} \rightharpoonup u$, $y_i-\overline{y_i} \rightharpoonup y_i$, and $ck_2\overline{u}-k_1\overline{y_1}=0$. The eigenvalue of this system is the root of the characteristic polynomial $\Psi(\rho)=det(\lambda\rho-A)$, where

$$A=\begin{pmatrix} -\frac{\overline{u}\lambda_1}{\overline{w}^2}u & -\frac{\overline{u}\lambda_1}{\overline{w}^2}u & \cdots & -\frac{\overline{u}\lambda_1}{\overline{w}^2}u \\ Ck_2 & -k_1 & \cdots & 0 \\ 0 & pk_1 & -k_1 & \cdots \\ \vdots & \vdots & \ddots & 0 \\ 0 & 0 & \cdots & -k_1 \end{pmatrix}_{(n+1)\times(n+1)}.$$

The determinant is calculated to obtain

$$\Psi(\rho)=\big(\rho+\frac{\lambda_1\overline{u}}{\overline{w}^2}\big)(\rho+k_1)^n+ck_2\frac{\lambda_1\overline{u}}{\overline{w}^2}\sum_{i=1}^{n}(\rho+k_1)^{n-1}(k_1p)^{i-1}.$$

From the characteristic polynomial with degree $n+1$, all the roots have negative real parts for $n=1,2,3$ by the Routh-Hurwitz stability criterion. The case of logistic growth is similar to that of the Simeoni growth.

References

1. Magni, P., Simeoni, M., Poggesi, I., Rocchetti, M. & De Nicolao, G. A mathematical model to study the effects of drugs administration on tumor growth dynamics. *Math. Biosci.* **200**, 127–151 (2006).
